# Expert viewpoint on endpoints in systemic sclerosis: current and future outlook

**DOI:** 10.1136/rmdopen-2026-006784

**Published:** 2026-07-15

**Authors:** Anna-Maria Hoffmann-Vold, Oliver Distler, Francesco Del Galdo, Jörg HW Distler, Yannick Allanore, Elana J Bernstein, Christopher P Denton, Masataka Kuwana, Marco Matucci-Cerinic, Margarida Alves, Vanessa Smith

**Affiliations:** 1 Department of Rheumatology, Oslo University Hospital, Oslo, Norway; 2 Department of Rheumatology, University Hospital Zurich, University of Zurich, Zurich, Switzerland; 3 Leeds Institute of Rheumatic and Musculoskeletal Medicine, University of Leeds and Biomedical Research Centre, Leeds Teaching Hospital Trusts, Leeds, UK; 4 Department of Rheumatology, University Hospital Düsseldorf, Heinrich-Heine University Düsseldorf, Düsseldorf, Germany; 5 Hiller Research Center, University Hospital Düsseldorf, Heinrich-Heine University Düsseldorf, Düsseldorf, Germany; 6 Department of Rheumatology, Cochin Hospital, Université Paris Cité, INSERM U1016, Paris, France; 7 Division of Rheumatology and Clinical Immunology, Department of Medicine, Vagelos College of Physicians and Surgeons, Columbia University Irving Medical Center, New York, New York, USA; 8 Division of Medicine, University College London, London, UK; 9 Department of Allergy and Rheumatology, Nippon Medical School Graduate School of Medicine, Tokyo, Japan; 10 Unit of Immunology, Rheumatology, Allergy and Rare Diseases (UnIRAR), IRCCS Ospedale San Raffaele Scientific Institute, Milan, Italy; 11 Vita-Salute San Raffaele University, Milan, Italy; 12 Inflammation Fibrosis and Ageing Initiative (INFLAGE), Division of Genetics and Cell Biology, IRCCS San Raffaele Scientific Institute, Milan, Italy; 13 TA Inflammation Med, Boehringer Ingelheim International GmbH, Ingelheim am Rhein, Germany; 14 Department of Rheumatology, Ghent University Hospital, Ghent, Belgium; 15 Department of Internal Medicine, Ghent University, Ghent, Belgium; 16 Unit for Molecular Immunology and Inflammation, VIB Inflammation Research Centre, Ghent, Belgium

**Keywords:** Biomarkers, Clinical Trial, Health-Related Quality Of Life, Patient Reported Outcome Measures, Scleroderma, Systemic

## Abstract

Systemic sclerosis (SSc) is a rare, heterogeneous, systemic autoimmune rheumatic disease characterised by vasculopathy, immune dysregulation and fibrosis affecting multiple organ systems. The complexity and variability of SSc manifestations pose significant challenges for clinical trial design and endpoint selection. Historically, three types of trial concepts have emerged: studies focused on a single organ; studies suggesting disease modification by combining a primary single-organ endpoint with supportive secondary endpoints across other systems, and studies explicitly designed to demonstrate disease modification through combined endpoints.

This expert review explores the current landscape and future directions of clinical endpoints in SSc trials. It highlights the growing importance of patient-reported outcomes, such as the Health Assessment Questionnaire-Disability Index and ScleroID, which provide valuable insights into patient experiences. The review also discusses the emergence of candidate composite endpoints, including the revised Composite Response Index in Systemic Sclerosis, which integrates clinical, functional and patient-centred measures to better reflect treatment efficacy.

Despite progress, significant unmet needs remain. Many endpoints assess damage at advanced stages, underscoring the need for earlier indicators of disease progression. The review advocates for incorporating time-to-event analyses, imaging and biomarker-based assessments to enhance sensitivity and relevance. It also emphasises the importance of refining composite endpoints to include continuous measures and broader domains such as vascular and gastrointestinal involvement. These efforts aim to improve the design and interpretability of SSc clinical trials, ultimately enhancing therapeutic development and patient outcomes.

**What is already known on this topic**

- Systemic sclerosis (SSc) is a complex autoimmune disease with diverse manifestations, which makes clinical trial design and selection of appropriate endpoints challenging. There is a need for endpoints that are more sensitive and can be used as earlier indicators of disease progression, including imaging measures and biomarkers.

**What this study adds**

- This expert review explores the current landscape of clinical endpoints in SSc, including the potential of emerging composite endpoints that integrate clinical, functional, patient-centred and time-to-event measures. It also emphasizes the importance of incorporating patient-reported outcomes to better capture how patients with SSc feel and function in clinical trials.

**How this study might affect research, practice or policy**

- These findings may support the development of more holistic and clinically meaningful endpoints for future SSc clinical trials.

## Introduction

 Systemic sclerosis (SSc) is a rare, heterogeneous, systemic autoimmune rheumatic disease characterised by three key features: vasculopathy, immune dysregulation, and fibrosis of the skin and internal organs.^1^ Due to the varying involvement of different organs and anatomical structures, patients present with a variety of clinical manifestations, such as Raynaud’s phenomenon (RP), skin tightening, digital ulcers, pulmonary arterial hypertension (PAH), interstitial lung disease (ILD), renal crisis, myocarditis, arthritis, gastro-oesophageal reflux disease (GERD), malabsorption and gastrointestinal (GI) motility dysfunction.^1 2^

Many early symptoms of SSc, such as RP, puffy fingers, GERD, fatigue and arthralgia, are non-specific and can overlap with other conditions,^1^ which makes diagnosis challenging. The initial presentation and the subsequent clinical manifestations that patients with SSc develop can be heterogeneous, variable and unpredictable.^1^ This multisystemic course highlights the complexity of SSc and the limitations of focusing on isolated symptoms or organ-specific complications when evaluating new therapies. To truly capture the systemic nature of SSc, there is a need to evolve the development and selection of endpoints in clinical trials.

## Treatment of SSc and assessing the effect of therapeutic interventions

The treatment of SSc should focus on addressing the underlying pathogenesis to reduce or halt disease progression, as well as managing its clinical manifestations. Various trials have evaluated the effects of immunosuppressants, antifibrotics, vasodilators and other drugs for treating SSc and its organ manifestations, such as ILD and PAH.^3^ Currently, no drug is approved worldwide specifically for the treatment of SSc.

Traditionally, outcome measures for assessing SSc report on single organ systems or single symptoms or their surrogates, including measures such as forced vital capacity (FVC) and the modified Rodnan skin score (mRSS).^4^ This has value as these organ manifestations are major causes of mortality and disease burden and/or are surrogates for poor long-term outcome of the overall disease. Beyond clinical trials, outcome measures are also important in observational studies and routine clinical practice, where they provide insights into the natural history and clinical diversity of SSc as well as helping to evaluate treatment effects. If combined as primary and secondary outcomes, these measures can give valid information regarding the effectiveness of treatments as disease-modifying agents. Given the importance of the patient’s perspective on their disease, these outcome measures should be complemented by or should incorporate patient-reported outcomes (PROs) such as the Health Assessment Questionnaire-Disability Index (HAQ-DI) and the Patient Global and Physician Global Visual Analogue Scale.^4^

Another strategy for assessing treatments in SSc is the development of multicomponent outcome measures. These include indices such as the revised Combined Response Index for Systemic Sclerosis (CRISS).^5^ These tools allow clinicians to evaluate the multifaceted benefits of treatments with one measure, especially those targeting organ systems most associated with morbidity and mortality.

This narrative review will provide expert insights into the unmet needs in clinical research regarding currently used endpoints, methods to capture disease progression and the potential to develop further composite endpoints—all issues recently raised by the European Medicines Agency as challenges for SSc clinical trial design.^6^ Although this review does not represent a systematic literature review and is not intended to be exhaustive, it reflects a viewpoint based on current expert opinion and available evidence. We envisage that this review may help guide the design of future clinical trials and improve the assessment of therapeutic interventions.

## Overview of current outcome measures in SSc clinical trials

There is currently a lack of consensus on how SSc clinical trials are designed and the efficacy endpoints used in these trials. Broadly, three trial concepts have emerged: (1) studies that target a single organ—such as RP, digital ulcers, ILD and PAH—using approved, organ-specific endpoints, a focus driven by the high burden of these manifestations in daily life, their association with increased mortality risk and further reinforced by regulatory endorsement of corresponding measures (eg, FVC for ILD, 6-minute walking test (6MWT) for PAH); (2) studies that claim disease modification by pairing a primary single-organ surrogate with positive secondary endpoints across other systems; and (3) studies that explicitly claim disease modification using combined indices (eg, revised CRISS). Notably, the phase of the trial appears to influence the selection of endpoints. Phase II proof-of-concept studies have typically focused on single-organ endpoints, such as FVC and mRSS, and have been conducted over shorter time periods. In contrast, current phase III registration trials are more likely to employ composite endpoints, as their aim is to demonstrate efficacy across multiple aspects of the disease in a broader patient population than has been included in previous earlier-phase studies.

Since around 2010, a core set of endpoints has been used most consistently as primary or key secondary outcome measures across multiple multicentre randomised clinical trials (RCTs). These endpoints form the focus of this section. Table 1 presents an overview of outcome measures that have been included as primary or key secondary endpoints in ≥3 RCTs in SSc (for an in-depth overview of outcome measures, refer to Chang and Pope^4^).

**Table 1:** (A) Diagnostic and technical outcome measures and (B) PROs used in SSc clinical trials*

(A)				
Outcome measure	Description	Strengths	Limitations	Example clinical trials
Skin				
mRSS	Measures skin thickness in 17 body areas	Simple, non-invasive, widely used	Inter-rater variability, spontaneous regression	DESIRES,^10^ faSScinate,^10^ focuSSced^10^
DU scores	Evaluates the severity and frequency of DUs	Can be assessed visually	Assessment of DUs with eschars, spontaneous healing	SEDUCE,^10^ DUAL-1,^10^ DUAL-2^10^
Lung				
FVC	Assesses lung function by measuring the amount of air a person can forcibly exhale; primary outcome measure in clinical trials for SSc-ILD	Non-invasive, predictive of mortality	Dependent on patient effort	SLS I,^10^ SLS II,^10^ RECITAL,^10^ SENSCIS^10^
6MWT	Assesses exercise capacity and endurance; primary outcome measure in clinical trials for PAH and SSc-PAH	Simple, predictive of mortality	Can be influenced by patient age, sex, height and weight and by (musculoskeletal) manifestations of SSc	PATENT-1,^10^ PATENT-2,^10^ ATPAHSS,^46^ STELLAR^47^
Time-to-event and survival				
Mortality-based endpoints	Reflects overall disease prognosis and treatment effect at the population level	Definitive, objective and clinically meaningful endpoint that reflects overall disease severity and treatment benefit across organ systems	Requires large sample sizes and long follow-up	GRIPHON,^48^ ZENITH,^7^ SLS II^10^
Time-to-event	Measure the time from a defined starting point until the occurrence of a specific event such as death, organ failure or disease progression	Captures dynamic change in disease, statistical efficiency	Many clinically meaningful events (eg, death, renal crisis) are infrequent in SSc	SERAPHIN,^49^ AMBITION,^22^ ASTIS^50^

(B)				
Outcome measure	Description	Strengths	Limitations	Example clinical trials
HAQ-DI	Evaluates the degree of difficulty a patient has in performing daily activities, providing a comprehensive measure of disability	Comprehensive and validated tool	Insensitive to organ‐specific changes (eg, lung) over short periods; ‘global’ nature may dilute detection of domain-specific benefit	VITALISScE,^51^ SENSCIS,^10^ PRedSS^52^
Patient and physician VAS	A simple scale completed by patients to rate overall disease severity, symptoms or global health (patient VAS), or by physicians to assess disease severity (physician VAS)	Simple to use; sensitive to changes in overall well-being or perceived disease activity; useful across diverse clinical settings and trial designs	Physician VAS may be affected by clinical experience and interobserver variability	ASSET,^53^ faSScinate,^10^ focuSSced^10^
UCLA SCTC GIT 2.0 questionnaire	Assesses GI symptoms and impact on quality of life	Non-invasive, patient-reported	Focused only on GI domain (not a global outcome measure)	ASSET,^53^ PROGASS,^10^ ReSScue^54^

* Used in at least three RCTs as primary or secondary endpoint.

AMBITION, phase III trial of ambrisentan and tadalafil in PAH; ASSET, phase II trial of abatacept in dcSSc; ASTIS, phase III trial of haematopoietic stem cell transplantation versus intravenous pulse cyclophosphamide in dcSSc; ATPAHSS, phase IV trial of tadalafil plus ambrisentan combination therapy in PAH; CRISS, Combined Response Index for Systemic Sclerosis; dcSSc, diffuse cutaneous systemic sclerosis; DESIRES, phase III trial of rituximab in SSc; DU, digital ulcer; DUAL-1/DUAL-2, phase III trials of macitentan in SSc; faSScinate, phase III trial of tocilizumab in SSc; focuSSced, phase III trial of tocilizumab in SSc; FVC, forced vital capacity; GI, gastrointestinal; GRIPHON, phase III trial of selexipag in PAH; HAQ-DI, Health Assessment Questionnaire-Disability Index; ILD, interstitial lung disease; mRSS, modified Rodnan skin score; 6MWT, 6-minute walk test; PAH, pulmonary arterial hypertension; PATENT-1/PATENT-2, phase III trial and open-label extension of riociguat in PAH; PRedSS, phase II trial of oral prednisolone in early dcSSc; PROGASS, open-label trail of prucalopride in SSc; RECITAL, phase II and III trials of rituximab versus cyclophosphamide in connective tissue disease-associated ILD; ReSScue, phase II trial of anaerobic cultivated human intestinal microbiome in SSc; SEDUCE, phase III trial of sildenafil in SSc; SENSCIS, phase III trial of nintedanib in SSc-ILD; SERAPHIN, phase III trial of macitentan in PAH; SLS I/SLS II, phase II trials of cyclophosphamide treatment and cyclophosphamide versus mycophenolate mofetil treatment in SSc-ILD; SSc, systemic sclerosis; SSc-ILD, systemic sclerosis-associated interstitial lung disease; SSc-PAH, systemic sclerosis-associated pulmonary arterial hypertension; STELLAR, phase III trial sotatercept plus background therapy in PAH; UCLA SCTC GIT 2.0, University of California Los Angeles Scleroderma Clinical Trials Consortium gastrointestinal tract instrument 2.0; VAS, visual analogue scale.

Overall, FVC and mRSS are now the most widely used endpoints for SSc as they evaluate relevant organ involvement (lung and skin involvement, respectively) through well-established measurement tools.^4^ For lung involvement, in SSc-ILD, FVC is the main outcome measure used in trials, often supported by additional functional and imaging measures such as diffusing capacity of the lung for carbon monoxide (DLco) and high-resolution CT (HRCT), respectively. The 6MWT has been used as a key outcome measure in clinical trials in PAH and SSc-PAH, reflecting exercise capacity and functional status.^7 8^ The mRSS is a surrogate tool to assess skin involvement in patients.^9^ Other domain-specific outcomes measures are discussed below.

### Digital ulcers

Digital ulcer scores are more commonly used in studies specifically targeting vascular complications of SSc. The phase III Sildenafil Effect on Digital Ulcer Healing in sClerodErma (SEDUCE) and phase III trials of macitentan in SSc (DUAL-1 and DUAL-2) randomised controlled trials all focused on evaluating treatments for digital ulcers in SSc. Each trial assessed either time to healing or the number of new ulcers as primary outcomes, reflecting disease activity and vascular involvement.^10^ Digital ulcer burden is another frequently used outcome measure.

### Gastrointestinal

GI manifestations currently lack outcome measures in SSc accepted by regulatory authorities, although efforts are underway to address this gap due to their substantial impact on patient quality of life (QoL). One tool specifically designed for GI involvement in SSc is the University of California, Los Angeles Scleroderma Clinical Trial Consortium Gastrointestinal Tract Instrument V.2.0 (UCLA GIT 2.0). This validated PRO measure assesses GI symptom severity and impact on daily functioning, and has seen increasing use in clinical research settings.^11^ However, a recent randomised trial using the UCLA GIT 2.0 as a primary endpoint failed to show a treatment effect.^12^

### Cardiac

There are currently no established cardiac endpoints for SSc. Cardiac involvement—measured using echocardiography and electrocardiograms (including 24-hour ECG Holter)—is assessed less frequently than other organ involvement. Myocarditis and myocardial fibrosis are increasingly reported in patients with SSc in clinical practice, likely due to improved physician awareness and advancements in non-invasive imaging technologies such as Doppler echocardiography and cardiac MRI.^13^ Although biomarkers like troponin T and N-terminal of the prohormone brain natriuretic peptide are widely used to assess cardiac involvement,^14^ they lack specificity for distinguishing between various cardiac and non-cardiac conditions, and should be interpreted alongside imaging and clinical findings. At present, there are no validated PROs specific for cardiac involvement in SSc, but symptom burden (eg, dyspnoea, palpitations, chest pain) can be captured through global or cardiovascular PROs used in related conditions. Despite the lack of established endpoints, cardiac assessment is important in clinical trials given that cardiac involvement is a major cause of death in SSc.^1^

### Musculoskeletal

There are also no dedicated outcome measures to assess musculoskeletal involvement for SSc, and thus researchers employ outcome measures commonly used in other rheumatic or autoimmune diseases.^15^ Musculoskeletal involvement—measured using clinical and radiographic examinations—remains an important area for exploration because of its implications for physical function. Tendon involvement is typically assessed through physical examination/functional tests and tendon friction rubs.^16^ Arthritis is evaluated using joint counts or inflammatory markers.^17^ A study validated the Disease Activity Score in 28 joints-Erythrocyte Sedimentation Rate (DAS28-ESR) and Disease Activity Score in 28 joints in C reactive protein, Simplified Disease Activity Index and Clinical Disease Activity Index for assessing arthritis in patients with SSc and found all to be valid measures with good reliability. Although challenges remain in detecting joint swelling in patients with advanced skin fibrosis, DAS28-ESR performed best in terms of reliability and construct validity, making it the most suitable tool among those tested.^15^ Although the assessment of muscle involvement or myositis is not standardised, a reasonable approach would be to use a combination of clinical, functional, laboratory and imaging evaluations routinely applied in clinical practice (such as assessing symptoms, HAQ-DI, elevated muscle enzymes, electromyography abnormalities and MRI). Of interest, the MYONET network study of patients with idiopathic inflammatory myopathies used core disease activity and damage indices, which may provide a useful framework for evaluating myositis.^18 19^ Notably, myopathy/myositis represents a key clinical feature in certain SSc subsets, such as U3RNP-positive patients, particularly in patients of African ancestry.^20^ There are currently no musculoskeletal PROs specific to SSc, although general instruments assessing pain and physical function are used.

### Renal

Renal involvement is measured in clinical trials, mainly for safety reasons, using blood tests to assess serum creatinine levels and estimated glomerular filtration rate, alongside monitoring blood pressure and urine analysis for proteinuria.^21^ There are currently no validated renal PROs in SSc.

### Mortality-based endpoints

Mortality endpoints are definitive, objective and clinically meaningful but their use can be challenging due to limited event rates and long follow-up requirements. For example, the phase III trial of ambrisentan and tadalafil in PAH (AMBITION) trial used time to clinical failure to capture treatment effects earlier in disease evolution.^22^ The phase III trial of haematopoietic stem cell transplantation versus intravenous pulse cyclophosphamide in diffuse cutaneous SSc (dcSSc; ASTIS) trial in SSc used time to death or major organ failure as its primary outcome.^10^

### Patient-reported outcomes

Use of PROs for global assessment is increasingly recognised by regulators, clinicians and patients as a valuable tool for collecting patient-centred data, and PROs are therefore increasingly included in clinical trials as primary or secondary endpoints to provide unique patient perspectives on the impact of a medical condition and its treatment.^23^ For SSc, PROs such as the HAQ-DI and ScleroID provide valuable insight into patient experiences related to pain, fatigue, disability and QoL, making them increasingly important in endpoint development.^24^ ScleroID, which represents the first comprehensive SSc-specific PRO measure, is of particular value given that it is disease specific, was patient derived and has been validated in a large European clinical cohort.^24^ The HAQ-DI has value as a generic instrument for assessing disability and health-related QoL, although it is not SSc specific. Relevant health domains for the HAQ-DI were chosen and prioritised by patients, resulting in the inclusion of 10 final domains.^21^ In addition, the Scleroderma Skin Patient-Reported Outcome (SSCPRO) is a validated, skin-specific PRO that has demonstrated sensitivity to change in a phase II study of lenabasum in dcSSc.^25^

Several measures are available to measure fatigue in SSc in clinical trials, including Functional Assessment of Chronic Illness Therapy-Fatigue (FACIT-F) and the Multidimensional Assessment of Fatigue.^26^ These are validated PROs assessing the severity and functional impact of fatigue, although they are not SSc-specific.

## Candidate multicomponent and composite outcome measures

There have been long-standing efforts towards developing comprehensive composite endpoints to better measure SSc disease activity. Multiorgan, composite measures are increasingly being applied to assess treatment efficacy in SSc clinical trials.^27^ Promising composite outcome measures that could be used in future SSc trials and their strengths/limitations are shown in table 2. These have been used to either assess disease damage, disease activity or to help stratify patients at risk of progression (stratification endpoints). Utility and validation of these dynamic endpoints in interventional trials will require further evaluation.

**Table 2:** (A) Diagnostic and technical outcome measures and (B) PROs for future SSc clinical trials

(A)				
Outcome measure	Measures included	Strengths	Limitations	Use
EUSTAR Disease Activity Index^55^	Skin, lung, renal, cardiac, musculoskeletal, laboratory markers	Easy to implement; measures activity, not damage	Not yet tested in RCTs	Predominantly applied in observational cohorts/registries to assess disease activity
SCTC Activity Index^56^	Skin, lung, GI, cardiac, renal, musculoskeletal, vascular, general symptoms (eg, pain)	Evaluates changes over previous 3–6 months; includes PROs	Complex; not anchored against other activity measures; not tested in RCTs	Applied in observational and development cohorts; proposed for trial enrichment and longitudinal monitoring^27^
SCTC Damage Index^57^	Skin, lung, cardiac, renal, GI, musculoskeletal, vascular, other organ damage	Very detailed assessment	Potential limitations in defining irreversible damage; complex; not tested in RCTs	Applied in observational cohorts; prognostic tool for damage accrual and mortality risk^27^
MINIMISE Combined endpoint^28^	SSc-related mortality, SSc-related manifestations (eg, new PAH, ILD progression)	Includes time-to-event endpoints for severe SSc; clinically meaningful	Only developed for use in trials; requires validation	Applied in observational study (assessing the value of serum type I interferon score in predicting clinically meaningful progression in limited cutaneous SSc)^28^
Revised CRISS^5^	Patient and clinician global assessments	Non-weighted contribution to overall score	Requires further validation of PRO weighting	Primary/Secondary outcome in RCT: DAISY^29^

(B)				
Outcome measure	Measures included	Strengths	Limitations	Use and example trials
ScleroID^24^	RP, hand function, fatigue, pain, skin, GI, respiratory, emotional well-being	Feasible to implement; developed with patients; covers all aspects of disease; validated	Awaiting feedback from regulators	Validation in observational STRIKE cohort (good correlation with clinical measures and PROs, and sensitivity to change over 12 months)^58^
COAST^31^	GI, lung, musculoskeletal, calcinosis, skin, hand function, fatigue, RP, DUs, other (appetite loss, weight change)	Robust SSc-specific, PRO measure for early SSc clinical trials	Still undergoing validation; regulatory acceptance not yet established	Preliminary questionnaires for use as outcome measures in clinical trials currently being assessed in an ongoing CONQUEST platform trial^32^
CRISTAL^33^	General domains (fatigue, sleep, pain, cognition), lcSSc-specific domains (RP, DUs, calcinosis, GI, joint/muscles, lung, sicca, skin)	Assesses the symptoms that are common and/or bothersome specifically in patients with lcSSc	Not yet validated in a longitudinal, multicentre, international cohort of patients with lcSSc	Aimed at the subset of patients with lcSSc and is intended for use in future clinical trials as a combined response index

COAST, Clinical Outcome Assessments for Systemic sclerosis clinical Trials; CONQUEST, phase II trial of amlitelimab and nerandomilast in SSc-ILD; CRISS, Composite Response Index in Systemic Sclerosis; CRISTAL, Combined Response Index for Scleroderma Trials Assessing lcSSc; DAISY, phase III trial of anifrolumab in SSc; DU, digital ulcer; EUSTAR, European Scleroderma Trials and Research group; GI, gastrointestinal; ILD, interstitial lung disease; lcSSc, limited cutaneous systemic sclerosis; MINIMISE, Multinational Investigation of Mortality and Manifestations in SSc Endpoint; PAH, pulmonary arterial hypertension; PRO, patient-reported outcome; RCT, randomised controlled trial; RP, Raynaud’s phenomenon; SclerolD, Scleroderma Impact of Disease index; SSc, systemic sclerosis; SSc-ILD, systemic sclerosis-associated interstitial lung disease; STRIKE, a retrospective cohort study on stratification for risk of progression in scleroderma.

The European Scleroderma Trials and Research group Disease Activity Index includes key clinical indicators such as worsening skin involvement, digital ulcers, elevated mRSS, tendon friction rubs, elevated C reactive protein and DLco <70% predicted, but lacks comprehensive cardiopulmonary and GI measures, and does not include PROs.^27^ The Scleroderma Clinical Trials Consortium (SCTC) Activity and Damage Indices provide structured assessments across multiple domains, offering a detailed evaluation of disease activity and irreversible organ damage. The SCTC Damage Index in particular helps identify patients at higher risk of progression.^27^ However, their complexity restricts ease of use in routine practice, and they do not incorporate PROs. In addition, the activity measures classify changes in damage as evidence of activity, which limits their value in cross-sectional assessments. Importantly, neither index has yet been validated as an outcome measure, and their responsiveness to improvement or worsening remains uncertain. Therefore, their reproducibility and correlation with other instruments require further validation.

The combined endpoint used in Multinational Investigation of Mortality and Manifestations in SSc endpoint (MINIMISE) trial is a novel time-to-event composite endpoint that includes SSc-related mortality, SSc-related organ manifestations (eg, new onset of PAH, new ILD or ILD progression), clinically significant worsening of mRSS, new onset of SSc-related cardiac disease, new digital ulcers requiring hospitalisation and significant GI tract disease.^28^ The endpoint was recently used in an observational study to demonstrate the value of serum type I interferon (IFN) score for predicting clinically meaningful disease progression in limited cutaneous SSc (lcSSc), highlighting the potential of type I IFN score to assess lcSSc disease activity and even enrich lcSSc clinical trials to achieve a higher probability of clinically meaningful events.^28^

The revised CRISS-25 has been developed and is being used in the phase III trial of anifrolumab in SSc (DAISY) trial, which will help provide validation for the use of the tool over 52 weeks.^29^ The revised CRISS includes five core measures: mRSS, FVC, HAQ-DI and PROs like patient global assessment of disease status (PtGA) and clinician global assessment of disease status (CGA). This measure captures key aspects of disease severity, functional ability, and both patient and clinician perspectives on overall disease status.^5^

ScleroID, a PRO specifically designed for SSc, is another valuable composite outcome measure that captures 10 patient-selected domains (RP, fatigue, hand function, pain, upper and lower GI symptoms, life modifications, body mobility, dyspnoea and digital ulcers).^24^ The Patient Self-Assessment of Skin Thickness in Upper Limb questionnaire is a potentially useful future PRO instrument as it assesses the impact of skin involvement on health-related QoL.^30^

The Clinical Outcome Assessments for Systemic sclerosis clinical Trials (COAST) measure is an SSc-specific endpoint developed as a PRO tool for use in early SSc clinical trials, with preliminary questionnaires currently being evaluated within the ongoing phase II trial of amlitelimab and nerandomilast in SSc-ILD (CONQUEST) platform study.^31 32^ The Combined Response Index for Scleroderma Trials Assessing lcSSc (CRISTAL) endpoint was designed to assess symptoms that are common and particularly bothersome in patients with lcSSc.^33^ However, neither COAST nor CRISTAL has yet undergone full validation.

### Incorporating other modalities

Advances in imaging and biomarkers could improve endpoint sensitivity by providing objective measures of disease progression and treatment response. Although not approved outcome measures for SSc, imaging modalities such as HRCT, cardiac MRI, positron emission tomography with new tracers (such as fibroblast activation protein inhibitor, which requires validation) and lung ultrasound can offer valuable structural and functional information.^34–36^ Imaging and biomarker-based outcome measures, including HRCT for ILD and inflammatory markers, could enhance the ability to monitor disease progression and treatment effects. Additionally, while cardiac MRI may not yet be widely represented in the literature, it could offer more informative assessments of inflammation and fibrosis.

The Digital Artery Volume Index has not been consistently used in clinical trials but it is a promising outcome measure of digital ulcer disease in SSc, due to its ability to non-invasively predict future digital ulcers and worsening PROs, potentially aiding patient enrichment and stratification.^37^ However, it still needs to be evaluated in clinical trials, and it requires imaging support.

Nailfold videocapillaroscopy (NVC) has emerged as a promising tool for predicting severe organ involvement and progression.^38^ A multicentre digital ulcer study demonstrated that capillary loss was associated with future development of digital ulcers.^38^ Another multicentre observational study with central imaging analysis found a ‘severe’ (active/late) NVC pattern to be associated with an increased likelihood of novel severe organ involvement and/or progression in all organ systems assessed. Of note, capillaroscopy has been able to predict skin progression and novel PAH and is correlated with ILD progression.^39 40^ These findings underscore the importance of standardised, longitudinal assessment of progression criteria in both observational cohorts and interventional trials.

The inclusion of imaging, laboratory parameters, clinician-reported outcomes, PROs and mortality could be considered as endpoints in future clinical trials. Laboratory markers, including inflammatory and soluble biomarkers, may serve as indicators of disease activity and treatment response, but further validation is required. The integration of the modalities discussed here into clinical trials is hindered by challenges related to standardisation, accessibility and regulatory approval.

## Unmet needs in clinical trials in SSc: endpoints

The majority of outcome measures used in clinical trials assess damage at a late stage in the development of SSc. There is a need to focus on measures earlier in disease development. The Very Early Diagnosis of Systemic Sclerosis (VEDOSS) concept highlights the importance of early disease detection.^41^ Although these patients are in a very early stage of SSc, they already exhibit certain disease features in several organ systems. Individuals exhibiting several of the ‘red flag’ VEDOSS features (RP, puffy fingers, antinuclear antibodies, SSc-specific antibodies, abnormal nailfold capillaroscopy) often meet the 2013 American College of Rheumatology/European League Against Rheumatism classification criteria for SSc at follow-up.^42^ It remains to be demonstrated whether existing outcome measures can adequately capture the subtle clinical manifestations characteristic of this early disease stage or whether research is needed for the development and validation of novel endpoints specifically adapted to early disease. Such endpoints should detect subclinical organ changes, early immunological and microvascular abnormalities and minimal patient-reported symptoms, while remaining clinically meaningful to patients, treating physicians and regulators.

There is a current lack of comprehensive endpoints in clinical trials that fully capture how patients feel and function in their daily life. While tools such as the revised CRISS do address aspects of patient experience, the overall approach remains fragmented, with different endpoints targeting specific domains. Emerging tools like ScleroID aim to address this gap by offering a more integrated view of PROs.

## Future goals of SSc

Our review discusses a number of the issues recently raised by the European Medicines Agency as challenges for SSc clinical trial design, including the importance of clear definitions for disease progression and modification, preventing new organ involvement, and establishing well-defined and effective endpoints. The future outlook for treatments and outcome measures in SSc emphasises the importance of accurately identifying disease progression and improvement. Various definitions of SSc disease progression have been used in observational studies and clinical trials. For instance, van Leeuwen *et al* define disease progression as worsening in one or more organ systems, initiation of immunosuppressive treatment or death.^43^ This approach involves annual evaluations of organ involvement, including the heart, lungs, skin and GI system, using standardised criteria to ensure accurate tracking over time. Another study used a definition of progression as new renal crisis, decrease of lung or heart function, new PAH or death.^44^ In an unselected, prospective multicentre cohort using a definition that included new or progressive involvement of the peripheral vasculature, lungs, heart, skin, GI tract, kidneys, musculoskeletal system or death at the 12-month or 24-month follow-up, a high proportion of patients (77%) met this criterion. However, organ-system complications without clearly established clinical meaningfulness were included.^39^

Looking forward, the prevention of new organ involvement remains a critical endpoint, particularly in trials targeting patients with early-stage disease. Future assessments of disease progression should include time-to-event analyses, where the event is either progression of any organ involvement or new internal organ involvement. The MINIMISE composite endpoint for time to clinical worsening, which included SSc-related mortality and SSc-related organ manifestations, provides an example of one such endpoint.^28^ The development and validation of such endpoints will aid in the development of targeted therapies. Establishing core domains of effectiveness could be driven by the SSc expert community to progress conversation with regulatory bodies.

Time to disease progression is largely missing from current clinical trials. The first step of the revised CRISS accounts for worsening or incident cases of any internal organ involvement and could offer a valuable starting point for developing disease progression endpoints for incorporation into SSc clinical trials.^5^ Additionally, survival endpoints should be considered as important endpoints in the context of patient QoL and prolonged survival. In a stem cell transplantation study, a global rank composite score was used, in which participants were ranked based on a hierarchy of clinical outcomes assessed at 54 months; these were death, event-free survival (survival without respiratory, renal or cardiac failure), FVC, HAQ-DI and mRSS. Participants were compared pairwise, and the global rank composite score was derived from these comparisons, demonstrating 67% of pairwise comparisons to favour transplantation.^45^

## Expert viewpoint

The authors of this review reached consensus on the need to continue prioritising ILD and skin involvement in SSc clinical trials for dcSSc, given the availability of validated measures, their sensitivity to change and their clinical significance. However, these aspects alone do not provide a complete picture of SSc. Including measures such as time-to-event endpoints, which have been successfully used in the approval of PAH treatments, could further enhance the relevance and regulatory acceptance of these endpoints.

Composite endpoints offer the possibility to provide a more holistic view of improvements in SSc. In particular, refinements to the revised CRISS would strengthen its position as a comprehensive composite endpoint and make it a valuable tool. The expert opinion of the authors of this review is to replace CGA and PtGA with the SSc-specific composite endpoint ScleroID, which would capture the patient viewpoint on pain, fatigue, lung function, hand function (including RP and digital ulcers) and GI involvement. Composite endpoints could be further strengthened through the inclusion of minimal clinically important difference-based thresholds and validation in clinical trials.
