# Supplementary material for: Expert viewpoint on endpoints in systemic sclerosis: current and future outlook
Source: RMD Open. 2026 Jul 15;12(3):e006784. doi: 10.1136/rmdopen-2026-006784 (PMC13374462; doi:10.1136/rmdopen-2026-006784)
Supplement: online supplemental file 1 [file rmdopen-12-3-s001.pdf]

1

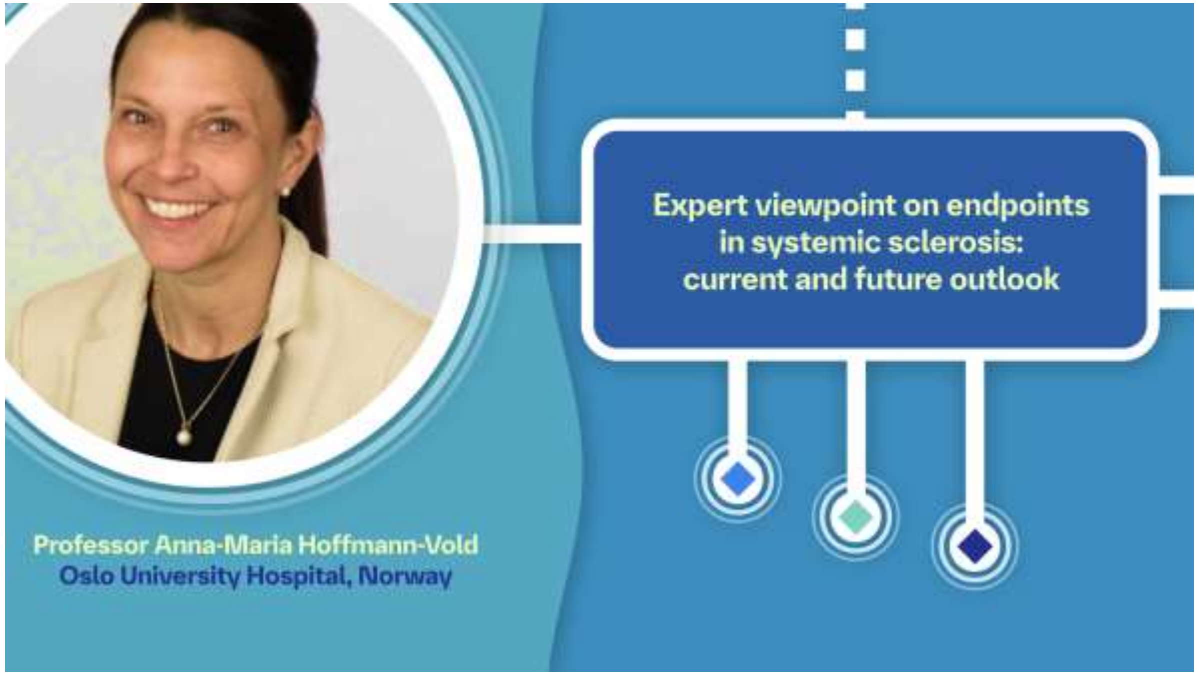

Script:
Hello, my name is Professor Anna-Maria Hoffman-Vold from Oslo University Hospital in Norway, and on behalf of my co-authors, I would like to present this short video on our review manuscript titled 'Expert viewpoint on endpoints in systemic sclerosis: current and future outlook'.

Animation notes:
Speaker circle appears, and lines emerge to form a flow diagram, with the title animating into the blue box. The speaker's name animates as she says it, and three endpoint icons appear as she says 'endpoints'.

2

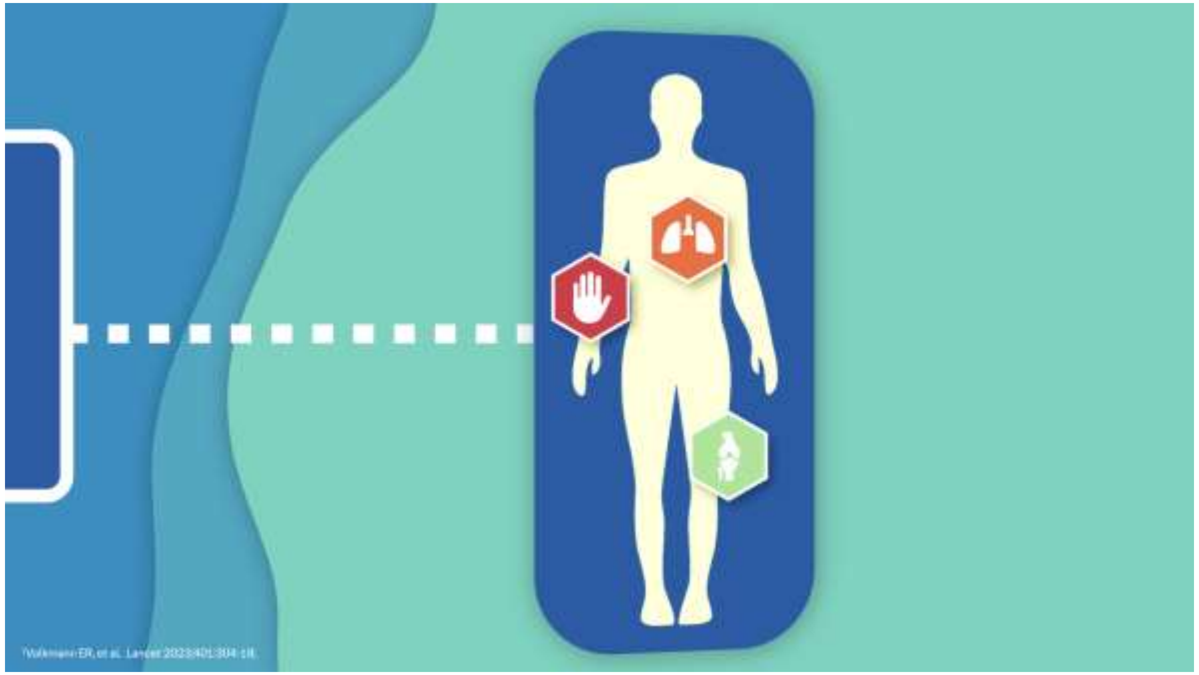

Script:
Systemic sclerosis, or SSc, is a rare autoimmune disease that affects multiple organs.

Animation notes:
The camera follows lines along to the right, and a panel appears containing a body diagram. Organ icons appear on the diagram.

3

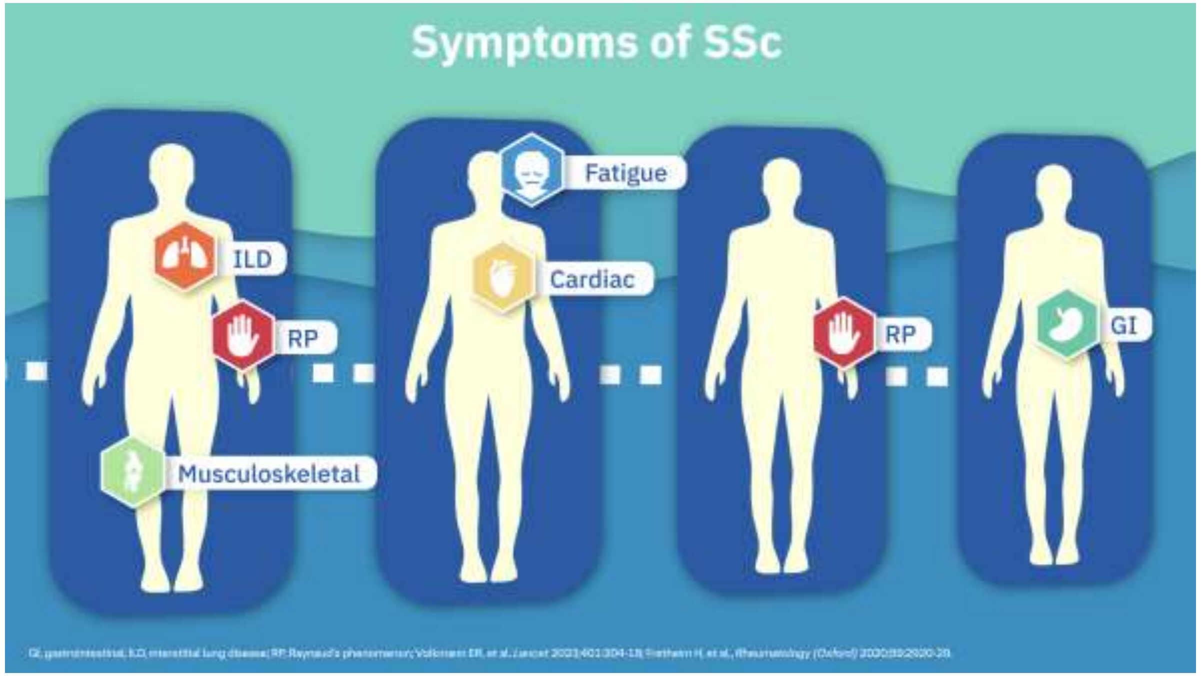

Script:
Because symptoms are complex and vary widely between patients, designing clinical trials with appropriate and meaningful endpoints is challenging.

Animation notes:
The camera keeps moving to the right, and more body panels appear, with different labels.

4

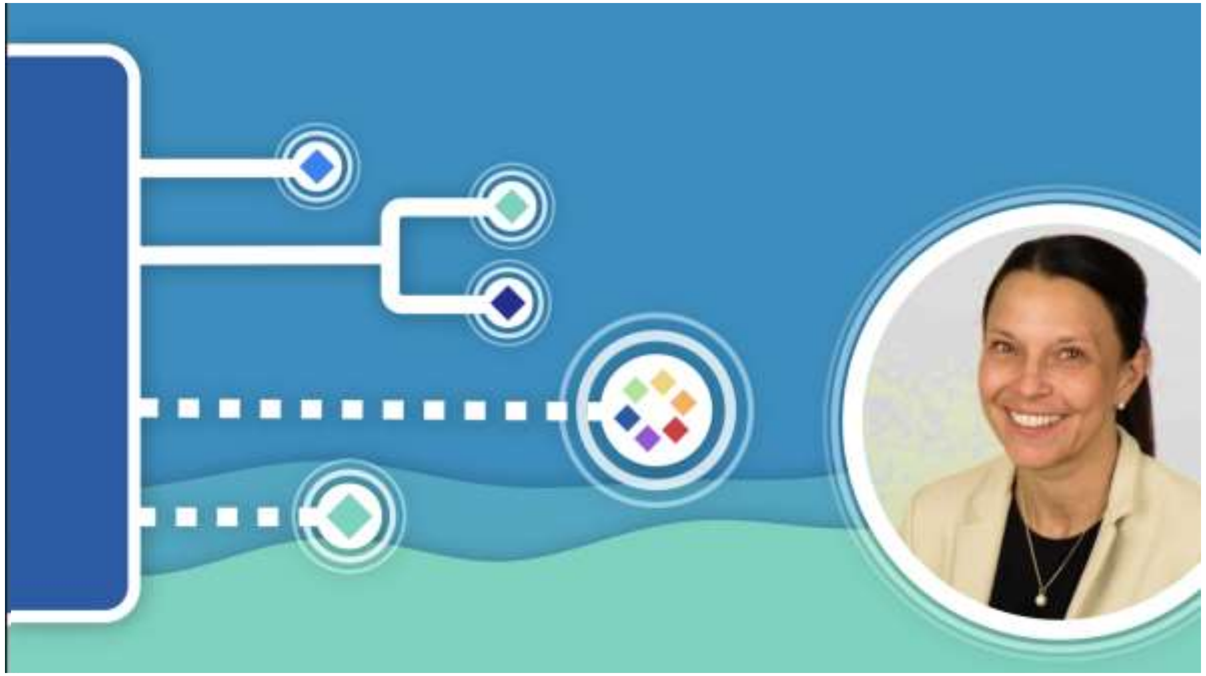

Script:
In our expert review, we examine the current endpoint landscape in SSc and discuss how endpoints may evolve to better support future clinical trials.

Animation notes:
The speaker circle reappears, and multiple lines terminate in different endpoints. These endpoints evolve, with the diamonds inside them changing colour and size.

5

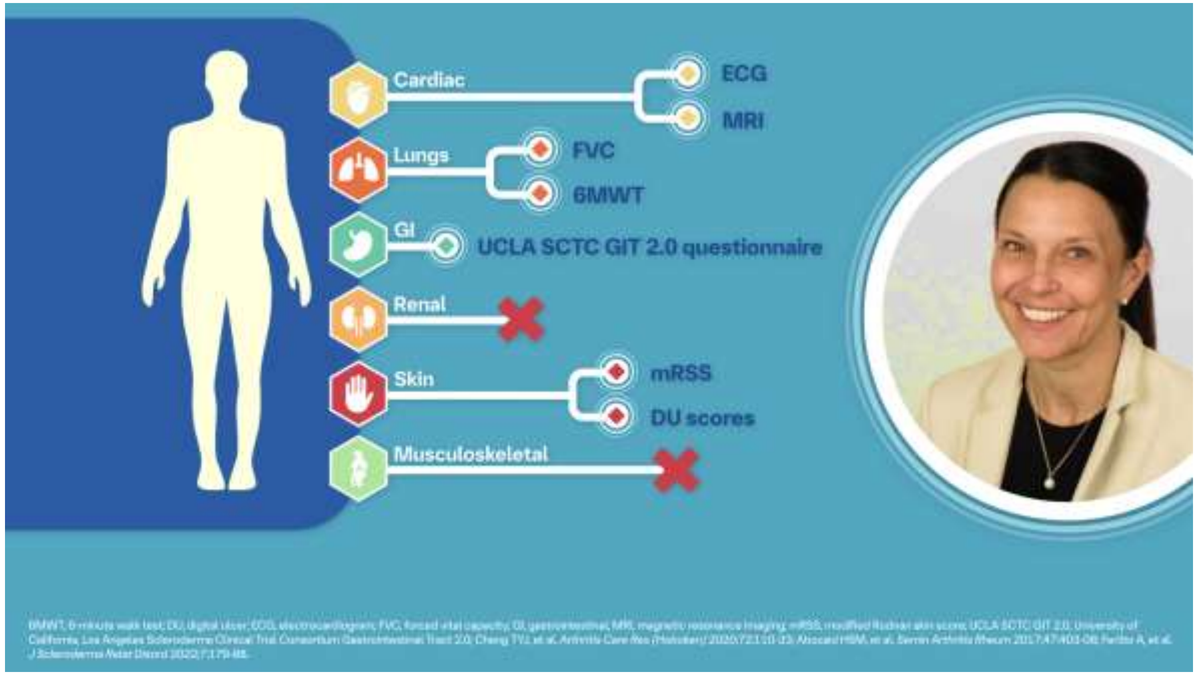

Script:
Traditionally, outcome measures in SSc trials have focused on single organs or symptoms. Examples include FVC for lung involvement, mRSS for skin disease, the UCLA SCTC GIT 2.0 questionnaire for gastrointestinal manifestations, and MRI for cardiac involvement. However, for many organ manifestations, there are still no established or approved endpoints in SSc clinical trials.

Animation notes:
A line emerges from each organ icon on the left, terminating at different endpoints (or red crosses), with labels appearing simultaneously.

6

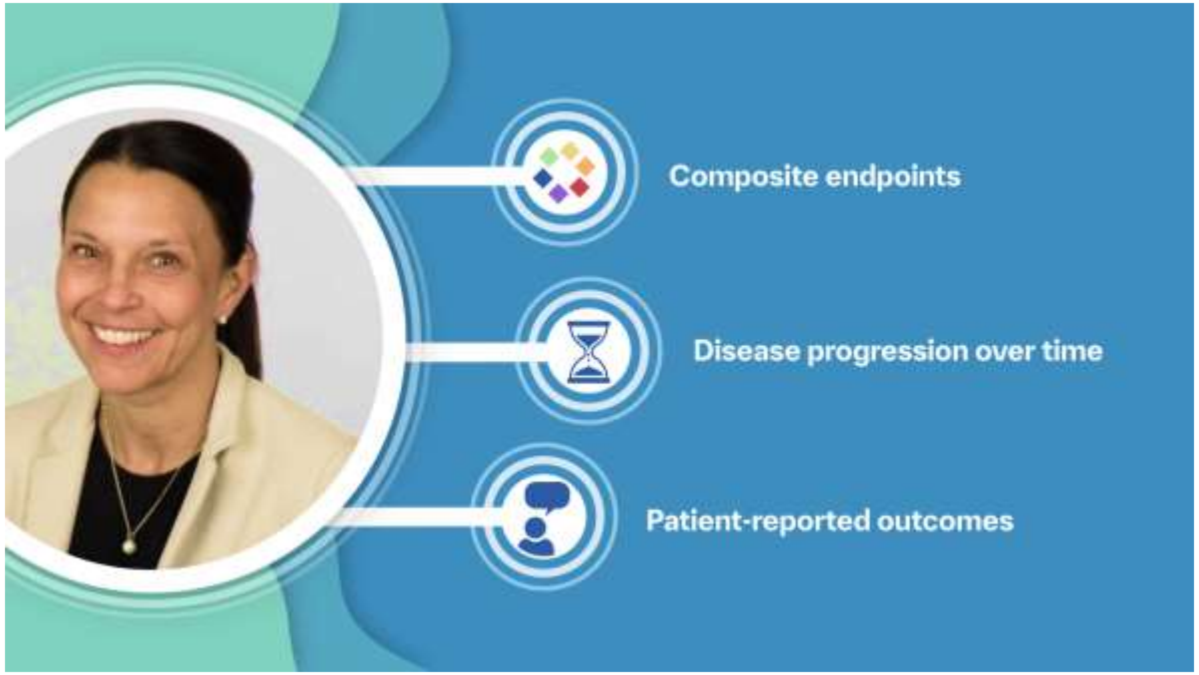

Script:
More recently, there has been increasing emphasis on endpoints that focus on three key areas: first, combining multiple domains to provide a more holistic assessment; second, measuring disease progression over time; and third, incorporating the patient's experience of SSc.

Animation notes:
The camera moves again. From the speaker circle, three additional endpoint icons appear, each with a label.

7

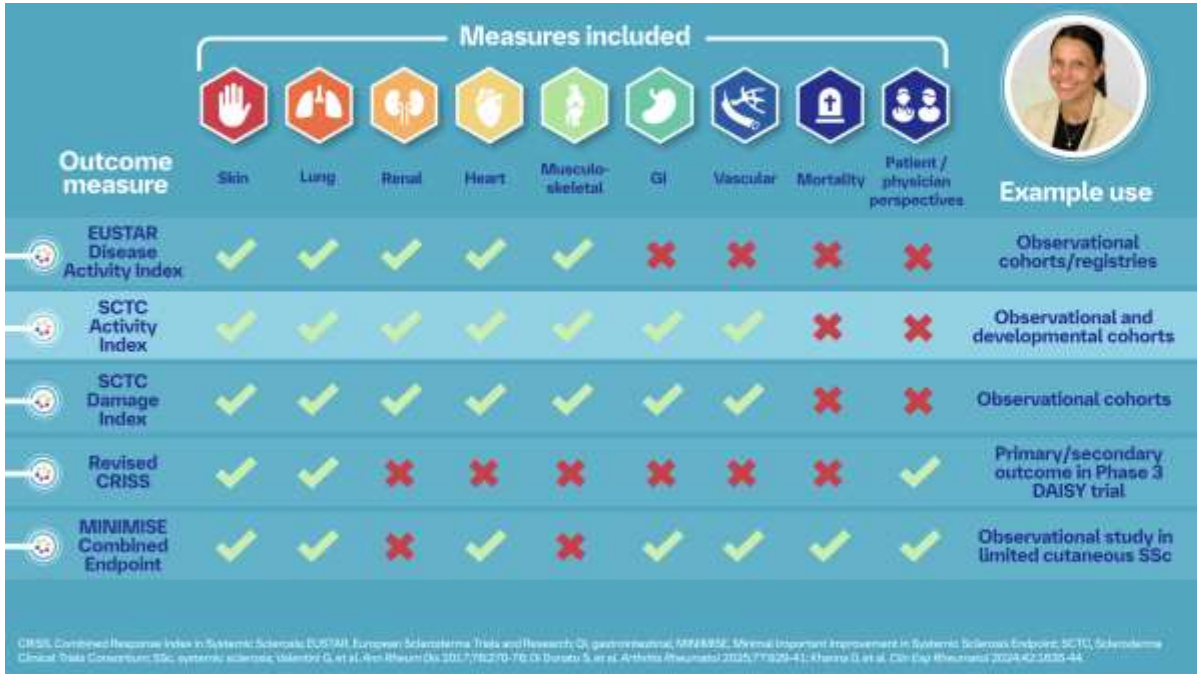

Script:
Multiorgan, composite measures can provide a more holistic view of improvements across multiple clinical, functional and patient-reported aspects of SSc. For this reason, they are increasingly being used in clinical trials. Tools such as the EUSTAR Disease Activity Index and the SCTC Activity and Damage Indices assess multiple clinical domains, although they have limitations, including complexity and limited inclusion of patient-reported outcomes. Newer composite endpoints, such as the revised CRISS-25 capture key aspects of disease severity, functional ability, and both patient and clinician perspectives on overall disease status, aiming to better capture clinically meaningful disease progression and patient-relevant change. The combined endpoint used in the MINIMISE trial is a novel

8

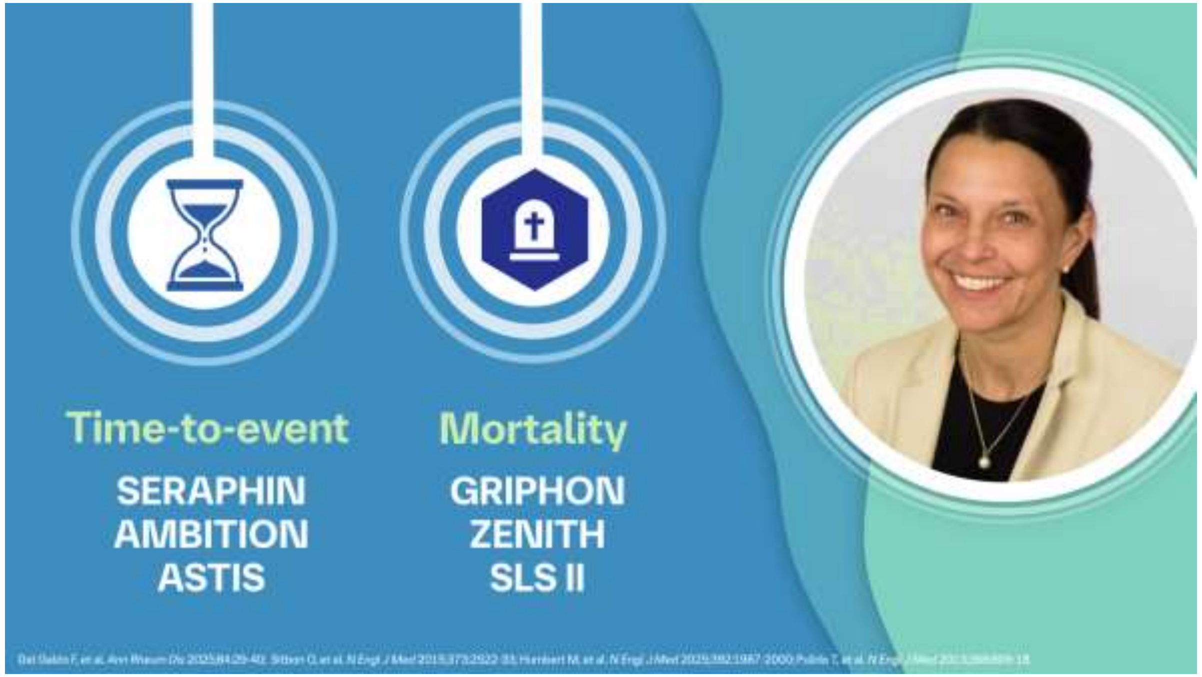

Script:
In addition to the MINIMISE endpoint, several other time-to-event endpoints have been developed and applied in clinical trials. Mortality endpoints, which are definitive and clinically meaningful, have also been used in multiple trials.

Animation notes:
For slide 7, the icons all appear along the top, the table forms, and rows are highlighted as the speaker mentions them. The table becomes populated with ticks and crosses.

For slide 8, the camera zooms towards the speaker, the table disappears, and two more endpoint icons appear at the end of the lines.

9

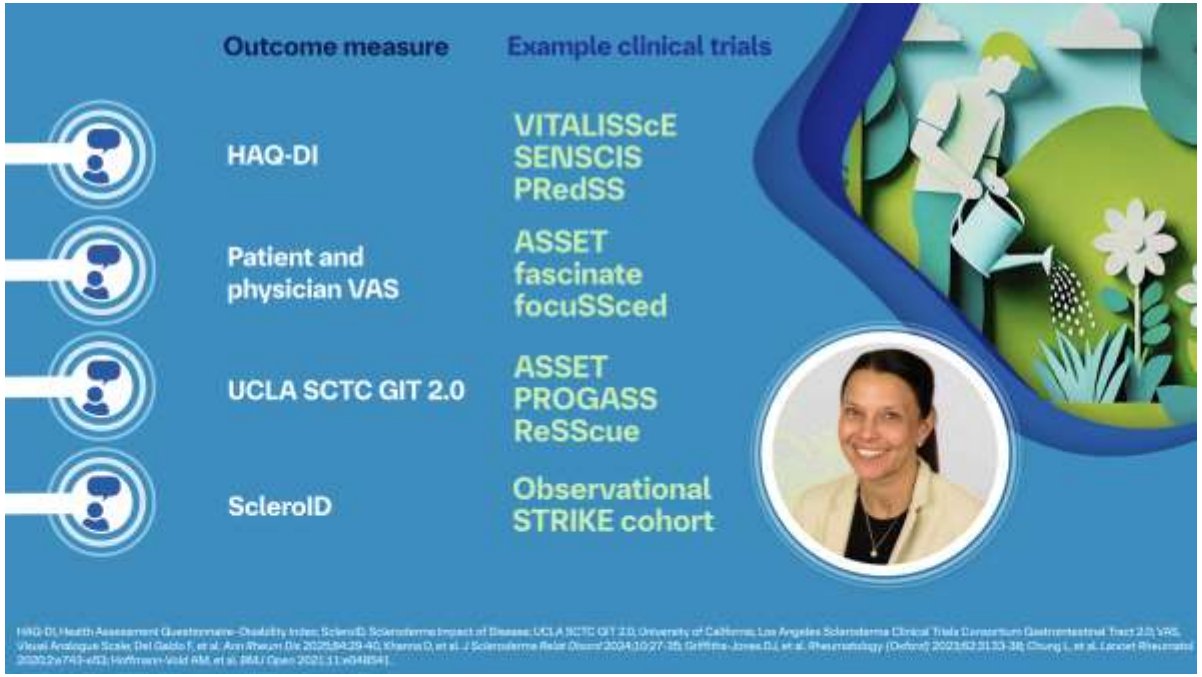

Script:
Patient-reported outcomes are increasingly used in clinical trials to help to provide a holistic view of the disease and how clinical changes affect patients' quality of life and daily functioning. Measures such as the HAQ-DI and ScleroID provide important insights into pain, fatigue, disability, and quality of life. ScleroID, in particular, is a validated, SSc-specific PRO developed directly with patient input, making it especially valuable.

Animation notes:
Four 'patient-reported' icons appear on the left, each joined by its label and examples of clinical trials. In the bottom right, several examples of characters enjoying a good quality of life are displayed one at a time, in a paper cut-out style. They are doing activities such as cooking, gardening, walking, shopping, etc.

10

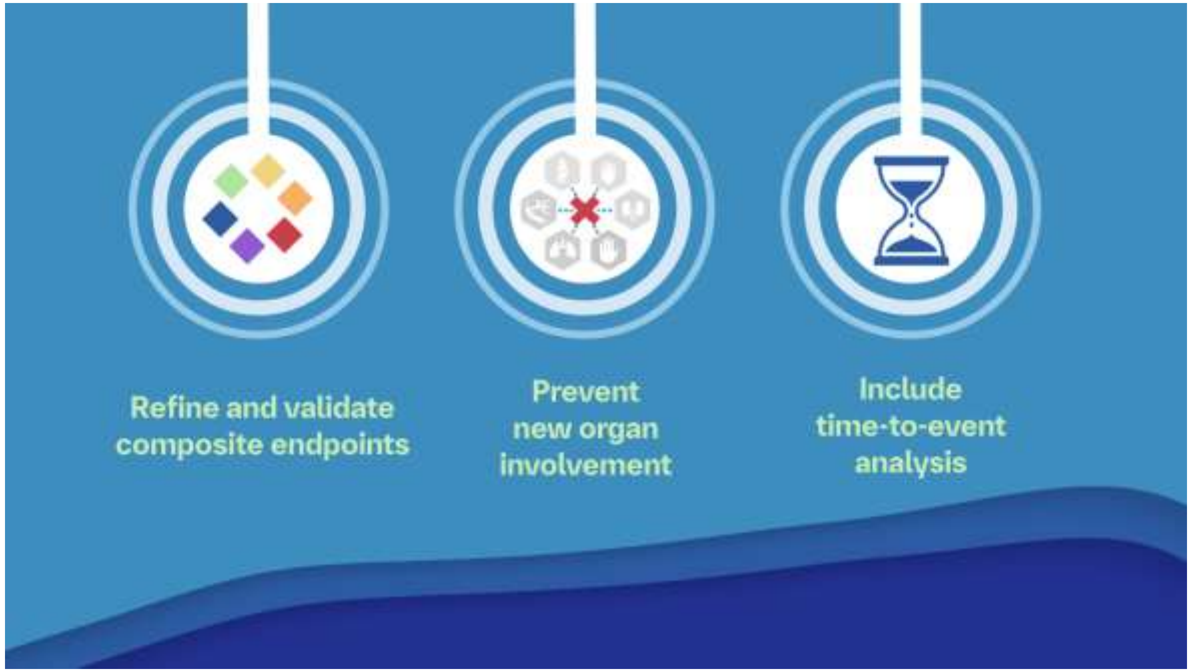

Script:
Looking forward, further refinement and validation of composite endpoints is needed. The prevention of new organ involvement remains a critical clinical endpoint, which is particularly important in trials targeting early-stage disease. Future assessments of disease progression should include time-to-event analyses, where the event is either progression of any organ involvement or new internal organ involvement.

Animation notes:
Three additional endpoint icons appear at the end of lines as they are mentioned in the script.

11

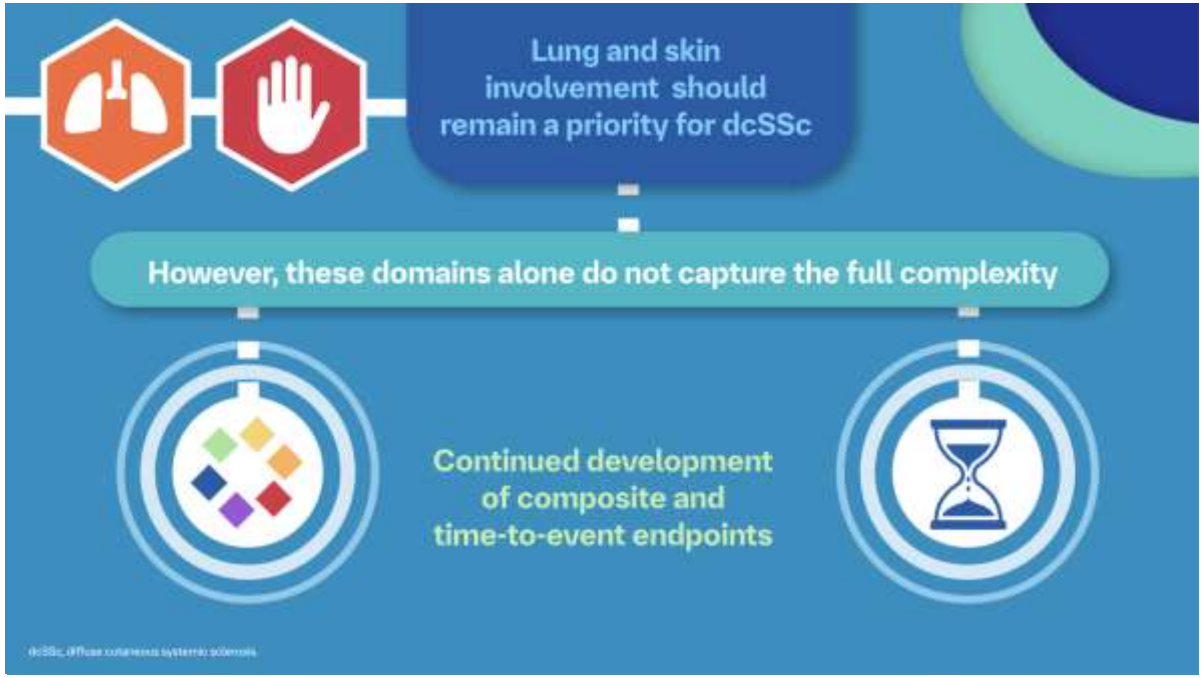

Script:
In conclusion, the authors of this review agree that lung and skin involvement should remain a priority due to their clinical importance and availability of validated measures. However, these domains alone do not capture the full complexity of SSc. Continued development of composite and time-to-event endpoints will be essential to better assess disease progression, support earlier detection of meaningful change, and advance future SSc clinical trials.

Animation notes:
A final section of the flow diagram imagery appears, with two organ icons appearing, and ending with the two large endpoint circles.

12

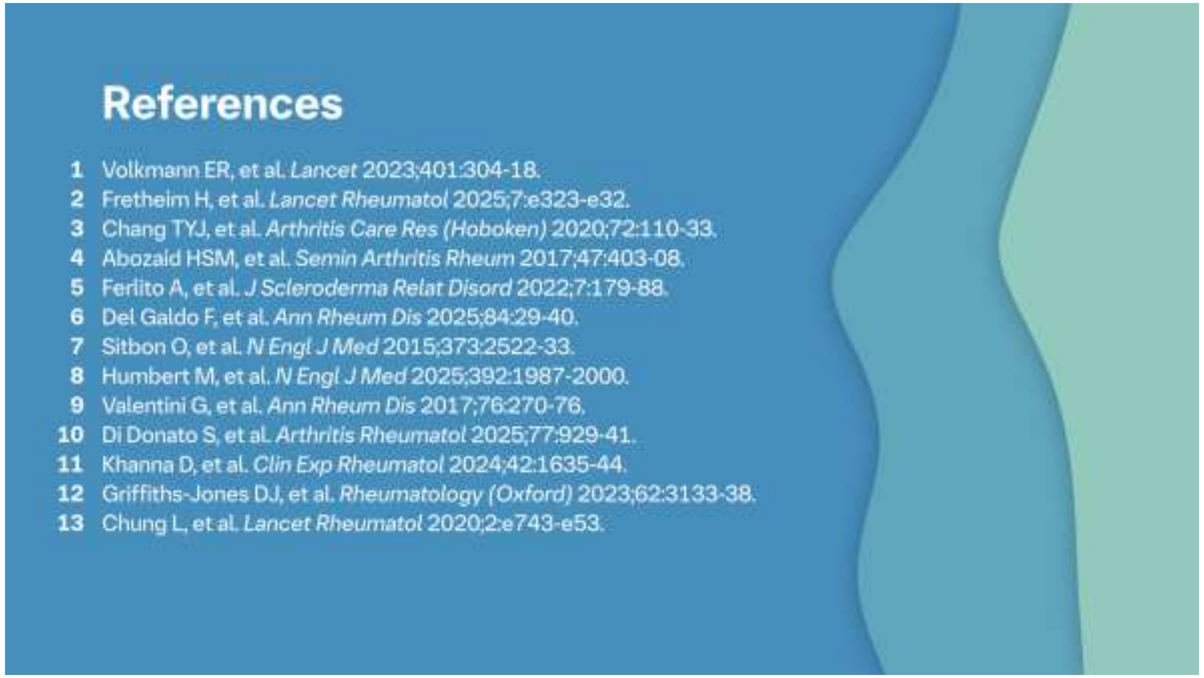

References

1. Volkmann ER, et al. *Lancet* 2023;401:304-18.
2. Fretheim H, et al. *Lancet Rheumatol* 2025;7:e323-e32.
3. Chang TYJ, et al. *Arthritis Care Res (Hoboken)* 2020;72:110-33.
4. Abozaid HSM, et al. *Semin Arthritis Rheum* 2017;47:403-08.
5. Ferlito A, et al. *J Scleroderma Relat Disord* 2022;7:179-88.
6. Del Galdo F, et al. *Ann Rheum Dis* 2025;84:29-40.
7. Sitbon O, et al. *N Engl J Med* 2015;373:2522-33.
8. Humbert M, et al. *N Engl J Med* 2025;392:1987-2000.
9. Valentini G, et al. *Ann Rheum Dis* 2017;76:270-76.
10. Di Donato S, et al. *Arthritis Rheumatol* 2025;77:929-41.
11. Khanna D, et al. *Clin Exp Rheumatol* 2024;42:1635-44.
12. Griffiths-Jones DJ, et al. *Rheumatology (Oxford)* 2023;62:3133-38.
13. Chung L, et al. *Lancet Rheumatol* 2020;2:e743-e53.

Animation notes:
The list of references appears.
